# Address by Dr. J. T. McMilen

**Published:** 1887-08

**Authors:** J. T. McMilen


### Address.

Before the Kentucky State Dental Association, June, 1887.

BY DR. J. T. MCMILEN.

Gentlemen of the Kentucky State Dental Association and Friends: It is to be hoped that this, our seventeenth annual meeting, will be blessed with harmony, a mutual endeavor and determination on the part of every one present, to lend his greatest energies to the advancement of the interests of our profession throughout this entire commonwealth.

It is hardly to be expected that I could suggest changes or formulate ideas anew, that had not been agitated in the minds of others, suggestions for the improvement and progress of our profession advanced by one, can only be made efficient by the co-operation of all.

Were it so, that we could get the attention, as well as the assistance from every man practicing dentistry in this State, in our attempt to elevate the standard of our profession, it would guarantee a better education of the people.

Educate the masses to appreciate the value of the best dental operations and we will, in a very short time, raise the standard of dental education in the profession. Wherever you find the people educated to the real necessities of dentistry, there you find good operations, and men who get and give a just value received for the services rendered.

Ignorant operators do not improve an ignorant public, but an ignorant public can be improved and educated by a scientific and properly educated operator.

It is unfortunate for our State that we have so few who take an active interest in this Association. It is lamentable to know the great number trying to practice dentistry whose every-day life is an imposition upon an unsuspecting and ignorant people. This condition of things can not be bettered in its present state, except by future amendments to our State law.

I fear that the last amendment made to our law, was not so worded as to secure the effect that was intended. While it has done well as far as it goes, it does not go far enough. I would call your attention to this amendment, section second.

There was, in the original draft of this section, two clauses which do not appear in the enactment as passed, which destroys the good it was intended to do. Whether this clause was dropped by accident, or was left out intentionally, to give the enactment a better constitutional aspect before the law, we have not learned.

The amendment in its present form has given the Board of Examiners an immense amount of work, and created considerable expense to this Association, for which we have received but little good. A code of ethics so arranged as would govern parties who have passed examination before a Board of Examiners of this Association, would be of great good to the dental fraternity throughout the State.

### OUR KENTUCKY DENTAL COLLEGE.

This addition to an already flourishing educational institution, is a step forward to bring our State (which has been so backward in its school system) up to a good standard. But do not let us make a mistake in this matter.

My friend, Dr. Smith, who has been the most active in the organization of a dental department to this institution, will not be held alone responsible for any mistake that might be made; nor will the faculty, or board of officers, or their associates of this College Association.

But, gentlemen, it will be the dental practitioners throughout the State that are responsible. The State Dental Association in connection with, and directed by, the State laws, should watch the organization and management of this dental school, as a parent would watch the growth, education, and prosperity of his children.

We can not be too careful in the selection of candidates for matriculation; we can not prepare them too well for a primary examination.

There is a tendency, all over this State, to hold out to young men too great inducements to undertake the study of dentistry. This inducement being for the paltry assistance the poor boy could render for a few months in handling the mallet, together with scrubbing the laboratory floor, and watching the vulcanizer; and with this he could get through college in one term, and do as good work, and get as good prices, as any dentist in Louisville.

Gentlemen, it should be a requirement that this college SHALL not matriculate a student unless said student had previously had, at least, two years' pupilage in the office of a dental practitioner qualified under our law.

We have lived to see Kenducky honored for her many virtues. Why not see her honored for the best dental college in the world? We have every facility for doing it.

Be guarded in the inducements you offer students; make them realize that success, in dentistry, is honesty toward all, with a true purpose, and persistant industry.

